# Multimodal MRI study of gray matter and functional connectivity abnormalities in adolescents with bipolar disorder

**DOI:** 10.3389/fpsyt.2025.1664729

**Published:** 2025-10-16

**Authors:** Pengyu Zhu, Yuxi Wang, Jialin Xiang, Junchen Gu, Xiong Chen, Fang Chen, Lulu Zou, Chunqi Ai, Kun Qin, Wen Chen

**Affiliations:** ^1^ Department of Radiology, Taihe Hospital, Hubei University of Medicine, Shiyan, China; ^2^ Mental Health Center, Taihe Hospital, Hubei University of Medicine, Shiyan, China

**Keywords:** bipolar disorder, resting-state functional magnetic resonance imaging, voxel- based morphometry, frontoparietal network, default mode network

## Abstract

**Background:**

Adolescent bipolar disorder (BD) is a severe psychiatric condition characterized by mood instability, with significant impacts on social and cognitive functioning. Clarifying the neural mechanisms underlying BD during adolescence may aid early diagnosis and treatment.

**Methods:**

We conducted a multimodal neuroimaging study integrating functional and structural MRI data to investigate alterations in spontaneous neural activity (amplitude of low-frequency fluctuation (ALFF) and regional homogeneity (ReHo)), seed-based resting-state functional connectivity (rsFC), and gray matter volume (GMV) in 69 adolescents with BD and 42 matched healthy controls (HCs). ALFF and ReHo were used to identify local functional abnormalities. Overlapping brain regions were selected as seeds for rsFC analysis. Voxel-based morphometry (VBM) was performed to detect GMV differences. Correlations between imaging measures and clinical symptom scores (HAMD, HAMA, YMRS) were assessed.

**Results:**

Compared to HCs, BD patients exhibited significant abnormalities in the ALFF within the default mode network (DMN) and the salience network (SN). ReHo was also altered in the SN. Seed-based rs-FC analysis revealed reduced connectivity between the right supramarginal gyrus and the left middle frontal gyrus, which are key nodes of the frontoparietal network (FPN). VBM analysis demonstrated decreased GMV in the left cerebellum. No significant correlations were found between imaging measures and clinical scale ratings.

**Conclusions:**

Our findings suggest that adolescent BD is characterized by functional abnormalities within DMN and FPN, as well as cerebellar gray matter atrophy. Disrupted structure–function coupling in these regions may reflect possible neurobiological mechanisms underlying BD during adolescence.

## 1 Introduction

Bipolar disorder (BD) is a chronic and debilitating mood disorder characterized by recurrent episodes of mania and depression, with a substantial risk of suicide and functional impairment (1). The disorder imposes considerable individual and societal burdens due to long-term cognitive and emotional dysfunction (2). It is estimated that the incidence rate of BD accounts for approximately 1-2% of the global population. Moreover, in recent years, the age of onset of BD has been continuously advancing (3), and the misdiagnosis rate of diagnosis has also been increasing, early misdiagnosis may miss the best treatment time. Therefore, it is of clinical importance to elucidate the early neurobiological mechanisms underlying adolescent BD and to inform timely and targeted interventions.

Recent advances in the field of neuroimaging have enhanced our understanding of the neurophysiology of this disease (4). In particular, the application of resting-state functional magnetic resonance imaging (rs-fMRI), abnormal local spontaneous brain activity, and resting-state functional connectivity (rsFC) have been repeatedly observed in patients with BD (5, 6). Previous studies have shown that abnormal pathways in large-scale functional networks may be the basis of the pathophysiology of BD (7, 8). In these studies, functional magnetic resonance imaging (fMRI) studies showed that compared with healthy controls (HC), patients with BD had abnormal resting-state functional connectivity (rsFC) in the default mode network (DMN), frontal-parietal network (FPN), salience network (SN), and limbic network (7, 8).

Alterations in gray matter volume (GMV) are also of critical interest in the context of BD, given their relevance to cognition, affect regulation, and disease progression (9). Gray matter structures, including the cerebral cortex and subcortical nuclei, are essential for perceptual, motor, and higher-order cognitive processes (10). Voxel-based morphometry (VBM) provides an automated, objective whole-brain technique to detect subtle morphometric changes associated with psychiatric and neurological disorders (11). Meta-analytic evidence indicates reduced GMV in the left orbitofrontal cortex, right putamen, and right dorsolateral prefrontal cortex in youth with BD relative to healthy controls (12).

Multimodal techniques combining structural and functional methods may provide more useful information for clinical diagnosis. For instance, combining the cortical thickness and functional connectivity (FC) analysis of MDD patients, Spati et al. found that there were indications suggesting that thinning of prefrontal cortex (PFC) might impair the participation of anterior cingulate cortex (ACC) during depressive episodes (13). van et al. found that the cortical thickness of dorsomedial prefrontal cortex (dmPFC) could be used to predict the rsFC between dmPFC and the default mode network (14). The combination of VBM and FC has also been used to study schizophrenia (15), temporal lobe epilepsy (16), and Parkinson’s disease (17).

In BD, emerging evidence suggests considerable neurobiological heterogeneity across clinical subgroups. Data-driven cognitive subtyping has revealed distinct patterns of structural and functional brain alterations, including prefrontal cortical thinning, white matter abnormalities, and altered activation/connectivity in frontoparietal circuits (2, 18–20). Furthermore, two studies demonstrated different BD subgroups based on neural activation during emotion regulation, characterized by differential activation of the prefrontal cortex and amygdala (11, 21, 22). Together, these findings suggest that there is a connection between the changes in brain structure and function in BD and clinical and cognitive phenotypes, which can serve as potential therapeutic targets.

Taken together, these findings highlight the interplay between structural and functional brain alterations and clinical phenotypes in BD, underscoring their potential utility as biomarkers for diagnosis and treatment stratification. However, reliance on unimodal imaging approaches may constrain integrative, transdiagnostic research, as emphasized in the Research Domain Criteria (RDoC) framework promoted by the U.S. National Institute of Mental Health. Combining neuroanatomical and functional connectivity analyses offers a promising avenue for delineating structure–function relationships and refining pathophysiological models of BD (23).

Therefore, the present study aims to investigate alterations in both brain structure and function among adolescents with BD and examine the associations between neuroimaging findings and clinical symptoms. This approach seeks to clarify the underlying neural mechanisms of adolescent BD, inform early identification strategies, and ultimately contribute to reducing diagnostic delays and errors in this vulnerable population.

## 2 Methods

### 2.1 Participants

A total of 111 adolescents were recruited for this study, including 69 patients with BD from Taihe Hospital, Hubei University of Medicine, and 42 HCs from the local community. Due to excessive head movement, seven subjects (five BD patients and two HCs) were excluded from the final analysis. All patients met the diagnostic criteria of DSM-IV structured clinical interview (SCID), but in this study, we did not distinguish between BD-I and BD-II. The exclusion criteria are: (1) age below 10 or above 20 years; (2) history of alcohol or substance abuse; (3) any current or past neurological or medical condition affecting cognitive function; (4) history of head trauma with loss of consciousness; (5) contraindications for MRI; and (6) any current DSM-5 Axis I psychiatric disorder other than BD. In this experiment, the manic symptoms of all patients were evaluated using the Young Mania Rating Scale (YMRS). The Hamilton Depression Scale (HAMD) is used to assess the depressive symptoms of patients, and the Hamilton Anxiety Rating Scale (HAMA) assesses the anxiety symptoms of patients. This research adheres to the Declaration of Helsinki. The current research has been approved by the Institutional Review Committee of Shiyan Taihe Hospital Affiliated to Hubei University of Medicine and Pharmacy. Written informed consent forms of all subjects were obtained before participating in this study.

### 2.2 fMRI data acquisition

All imaging data were collected using the GE 3T scanner at the Medical Imaging Center of Taihe Hospital in Shiyan City. Initially, the resting-state functional images were collected, followed by the T1 images. During the scanning process, the subjects were asked to try not to swallow or move their bodies, especially their heads. In the resting state, the subjects were also instructed to close their eyes, relax and stay awake. The resting-state functional images were collected using the echo planar imaging (EPI) sequence as follows: Axial scanning, repetition time (TR) = 2000ms, echo time (TE) = 30ms, flip angle = 52°, field of view (FOV) = 240 × 240 mm^2^, matrix size = 80×80, number of layers = 62, layer thickness = 3.5 mm Voxel size = 2 × 2 × 2 mm³. T1 images were collected using the brain volume (BRAVO) sequence prepared by T1-weighted sagittal 3D magnetization, as shown below: TR = 7.4ms, TE = 30ms, flip angle = 12°, FOV = 256 × 256 mm^2^, matrix size = 256 × 256, number of layers = 166, layer thickness = 1 mm, voxel size = 1 × 1 × 1 mm^3^.

### 2.3 fMRI data preprocessing

Resting-state fMRI data were preprocessed in the toolbox of (resting-state) brain imaging data processing and Analysis (DPABI v3.1) (24). The preprocessing steps include deleting the first five time points, slice timing, realignment, de-trend, removing the head movement effect (using the Friston 24-parameter model) (25), regression of nuisance covariates (white matter signal, cerebrospinal fluid signal, and global mean signal), normalizing the MNI space (using T1 images), and resampling (voxel size) = 2 × 2 × 2 mm3, spatial smoothing (6 mm full-width at half-maximum, applied to ALFF and rsFC analyses) and bandpass filtering (0.01-0.1 Hz, only applicable to ReHo and rsFC analysis). Furthermore, we use volume-based framewise displacement (FD) to quantify head movement (26). FD reflects the head movement from one volume to the next, and a volume with FD < 0.2 indicates a good point in time. The mean FD is calculated as the average of the sum of the absolute values of the differential realignment estimates at each time point (through backward differences) (27). If the number of good time points for the subjects is less than 160, the subjects will be excluded.

### 2.4 ALFF and ReHo calculation

Using the preprocessed resting-state fMRI data, we calculated the ALFF and ReHo values of each subject in DPABI v3.1. All values are calculated at the voxel level. Calculate the ALFF value using the signal strength in the low-frequency range (i.e., 0.01-0.1 Hz). The ReHo value, also called the Kendall’s coefficient of concordance (KCC), of the time series of a given voxel with its nearest neighbors (27 voxels were considered) was calculated to generate individual ReHo maps. Finally, the ALFF and ReHo maps of each subject were respectively converted into Z-score maps.

### 2.5 Functional connectivity analysis

This analysis was carried out in DPABI v3.1. The overlapping clusters exhibiting concurrent abnormalities in both ALFF and ReHo among patients with BD were selected as seeds for subsequent functional connectivity analysis. Specifically, clusters showing significant ALFF and ReHo alterations were first extracted to create masks, and their spatial overlap was identified as the seed regions. The average time series of the seed regions were extracted for each subject. Next, Pearson correlation coefficients were computed between the seed time series and the time series of every other voxel in the brain, resulting in a rsFC map for each individual. Finally, each subject’s rsFC map was converted into a z-score map using Fisher’s z-transformation to improve normality for further statistical analyses.

### 2.6 VBM analysis

We used the VBM8(http://dbm.neuro.uni-jena.de/vbm8) toolbox in SPM12(http://www.fil.ion.ucl.ac.uk/spm) software to analyze the T1-weighted images. First, check for artifacts in the image and adjust the source of the image to the front merge. Subsequently, the images were segmented into gray matter, white matter and cerebrospinal fluid maps, and then normalized to the standard Montreal Neuroscience Institute (MNI) space, with a voxel size of 1.5 × 1.5 × 1.5 mm³. After checking the data quality of the partitioned map, the gray matter map was further smoothed using an 8 mm full-width, semi-Gaussian kernel.

### 2.7 Statistical analyses

For the categorical variables of demographic and clinical data, we used chi-square tests for group comparisons. For continuous variables, we first performed a Shapiro–Wilk test to assess normality. If the variables follow a normal distribution, we use the two-sample t-test for group comparison. If the normal distribution is not followed, the Mann-Whitney U test is used to compare the differences between BD patients and HC patients. All statistical analyses were conducted using SPSS version 20.0.

For each functional measure (namely ALFF, ReHo and rsFC), the differences between the two groups were analyzed in SPM12. In these analyses, the two-sample t-test was used to control for age, gender and mean FD. Significance was determined using a voxel-level threshold of p < 0.001 (uncorrected) combined with cluster-level family-wise error (FWE) correction at p < 0.05. To further examine associations between imaging markers and clinical symptoms, we performed Pearson correlation analyses between ALFF and ReHo values from significant clusters and clinical scores (HAMD, HAMA, and YMRS). A Bonferroni-corrected significance threshold of p < 0.017 (0.05/3) was applied.

For GMV, group differences in gray matter volume were examined using two-sample t-tests in SPM12, controlling for age, sex, and total intracranial volume. The significance level was set as a combination of uncorrected P < 0.001 at the voxel level and FWE correction P < 0.05 at the cluster level.

## 3 Results

### 3.1 Demographic and clinical characteristics

**Table 1** summarizes the demographic and clinical data of all the subjects. We found that age did not follow a normal distribution (P < 0.05). Therefore, the median and the upper and lower quartiles are used for representation. There was a significant difference in gender distribution between the BD group and the HC group (χ² = 8.651, p = 0.003), among which the proportion of females was higher in the BD group. There was no significant difference in age between the two groups. (**Table 1**) Given the differences in gender distribution between the two groups (40.8% male in the BD group and 57.1% male in the HC group), gender was included as an independent covariate in the model in all secondary analyses to control its potential confounding effects. Meanwhile, age was also included in the analysis as a covariate (**Table 1**).

**Table 1:** Demographic and clinical feature of participants.

Demographic	Bipolar disorder (N = 69)	HC (N = 42)	Statistics	*P*
Age	15(14, 16)	15.8(12, 19)	-0.977(Z)	0.329
Sex (male/female)	20/49	24/18	8.651(χ2)	0.003
Age of onset	13.47 ± 1.638	NA	NA	NA
Duration of illness(m)	16.23 ± 14.571	NA	NA	NA
HAMD	30.5 ± 15	NA	NA	NA
HAMA	21.58 ± 10.5	NA	NA	NA
YMRS	14.6 ± 7.68	NA	NA	NA
Psychotic symptoms	58^a^	NA	NA	NA
Physical symptoms	59^b^	NA	NA	NA
Suicide consciousness	53^c^	NA	NA	NA
Antipsychotic drugs	30^d^	NA	NA	NA

NA, not applicable; HAMD, Hamilton Depression Scale; HAMA, Hamilton Anxiety Rating Scale; YMRS, Young Mania Rating Scale. ^abcd^Representing the corresponding number of people.

### 3.2 ALFF and ReHo analysis

**Figure 1:**
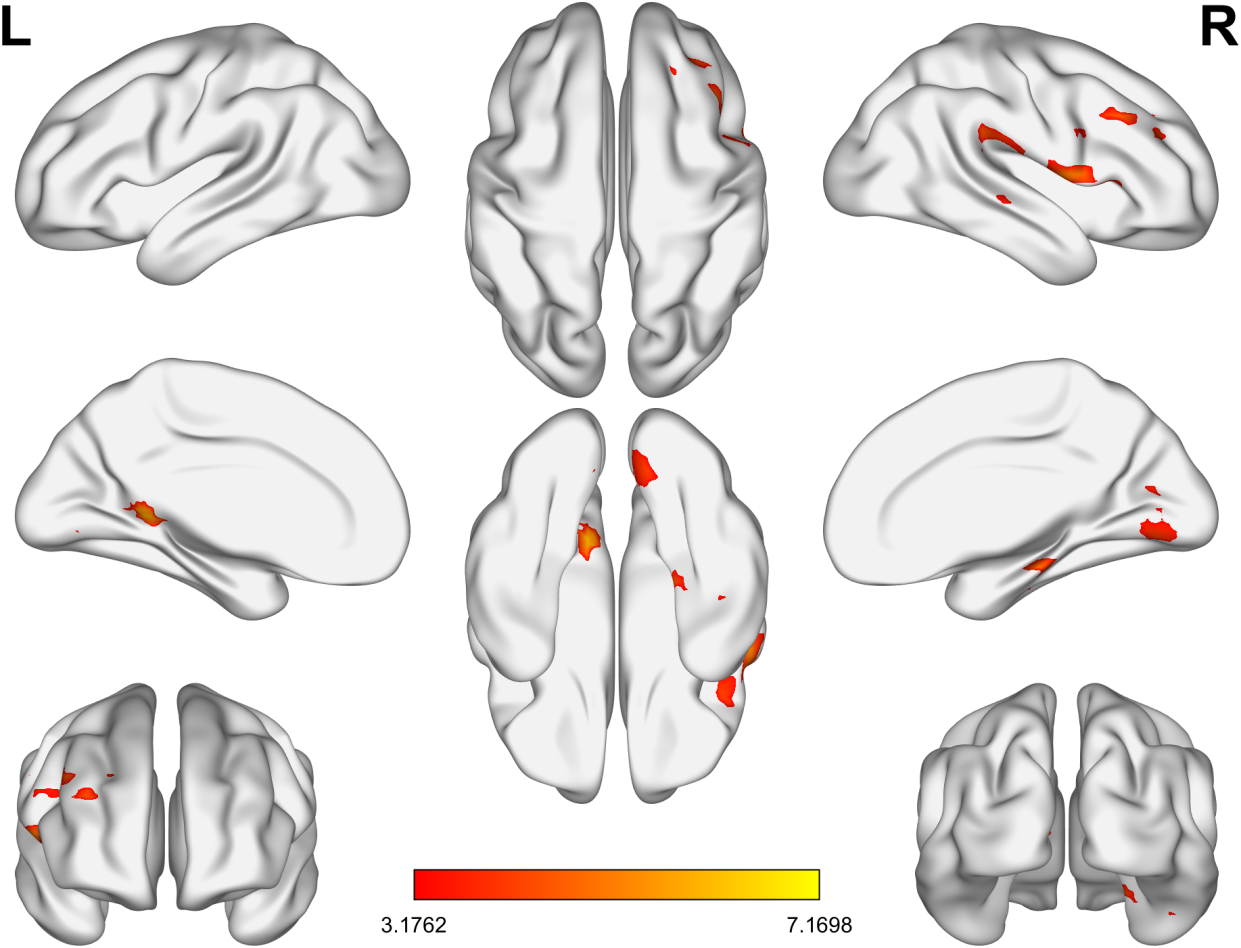
The brain regions in the BD group with significantly lower ALFF values than the HC group. BD, bipolar disorder; ALFF, amplitude of low-frequency fluctuation; HC, healthy control.

Whole brain analysis revealed that in the BD group, the ALFF values in the right supramarginal gyrus, right lingual gyrus, right middle frontal gyrus, left posterior cingulate gyrus, and right parahippocampal gyrus were significantly increased (**Table 2**, **Figure 1**).

**Table 2:** Between-group differences in the ALFF analyses between the patients with BD and the healthy controls.

Brain region	Hemisphere	MNI coordinates	Peak T values	Cluster size	Cluster-level *P*_FWE_
Patients >Controls					
Supramarginal gyrus	Right	48,-21,27	6.1856	468	<0.001
Lingual Gyrus	Right	6,-69,-3	4.8764	281	0.001
Middle Frontal Gyrus	Right	30,36,24	5.504	265	0.001
Posterior cingulate gyrus	Left	-12,-45,6	6.2056	200	0.003
Parahippocampal Gyrus	Right	15,-12,-15	7.1698	176	0.021

BD, bipolar disorder; ALFF, Amplitude of low-frequency fluctuation; MNI, Montreal Neurological Institute.

**Figure 2:**
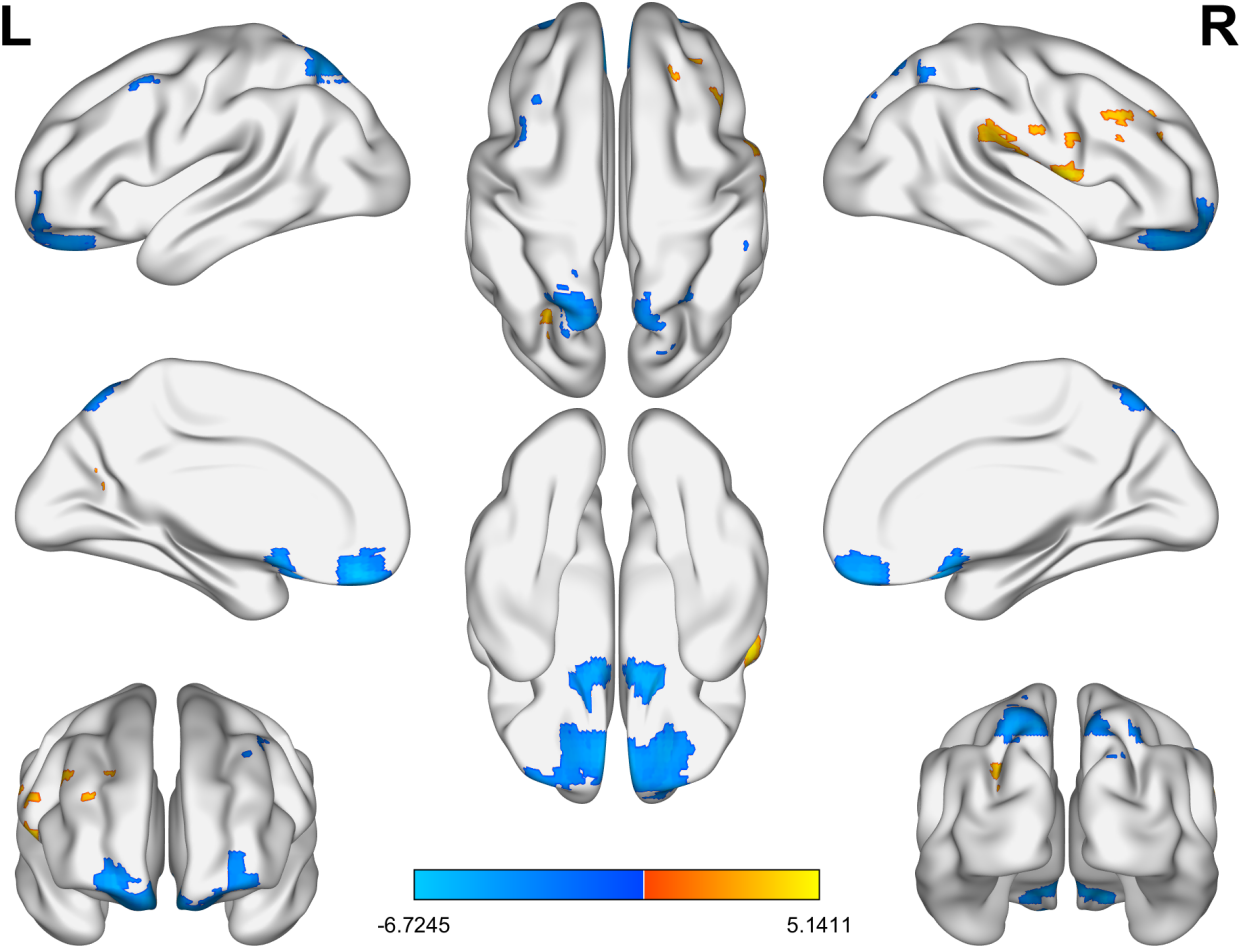
The brain regions with significant differences in ReHo values between the BD group and the HC group. Yellow indicates a significant increase and blue indicates a significant decrease. BD, bipolar disorder; ReHo, regional homogeneity; HC, healthy control.

As for ReHo, in the BD group, the ReHo values of the right supramarginal gyrus and the left superior occipital gyrus were significantly increased, while those of the left superior frontal gyrus, the left parietal gyrus, the right parietal gyrus, the right middle frontal gyrus and the right middle temporal gyrus were significantly decreased (**Table 3**, **Figure 2**).

**Table 3:** Between-group differences in the ReHo analyses between the patients with BD and the healthy controls.

Brain region	Hemisphere	MNI coordinates	Peak T values	Cluster size	Cluster-level *P*_FWE_
Patients >Controls					
Supramarginal gyrus	Right	48,-21,27	5.1411	229	0.001
Superior occipital gyrus	Left	-21,-66,21	4.4698	89	0.001
Patients <Controls						
Superior Frontal yrus	Left	-3,33,-27	6.7245	813	<0.001
Superior parietal gyrus	Left	-48,-45,51	5.4273	795	<0.001
Superior parietal gyrus	Right	39,-63,51	5.8941	517	<0.001
Middle frontal gyrus	Right	45,24,48	4.9873	300	<0.001
Inferior temporal gyrus	Right	57,-60,-15	6.0793	175	0.003

BD, bipolar disorder; ReHo, regional homogeneity; MNI, Montreal Neurological Institute.

### 3.3 Seed-based rsFC analysis

**Figure 3:**
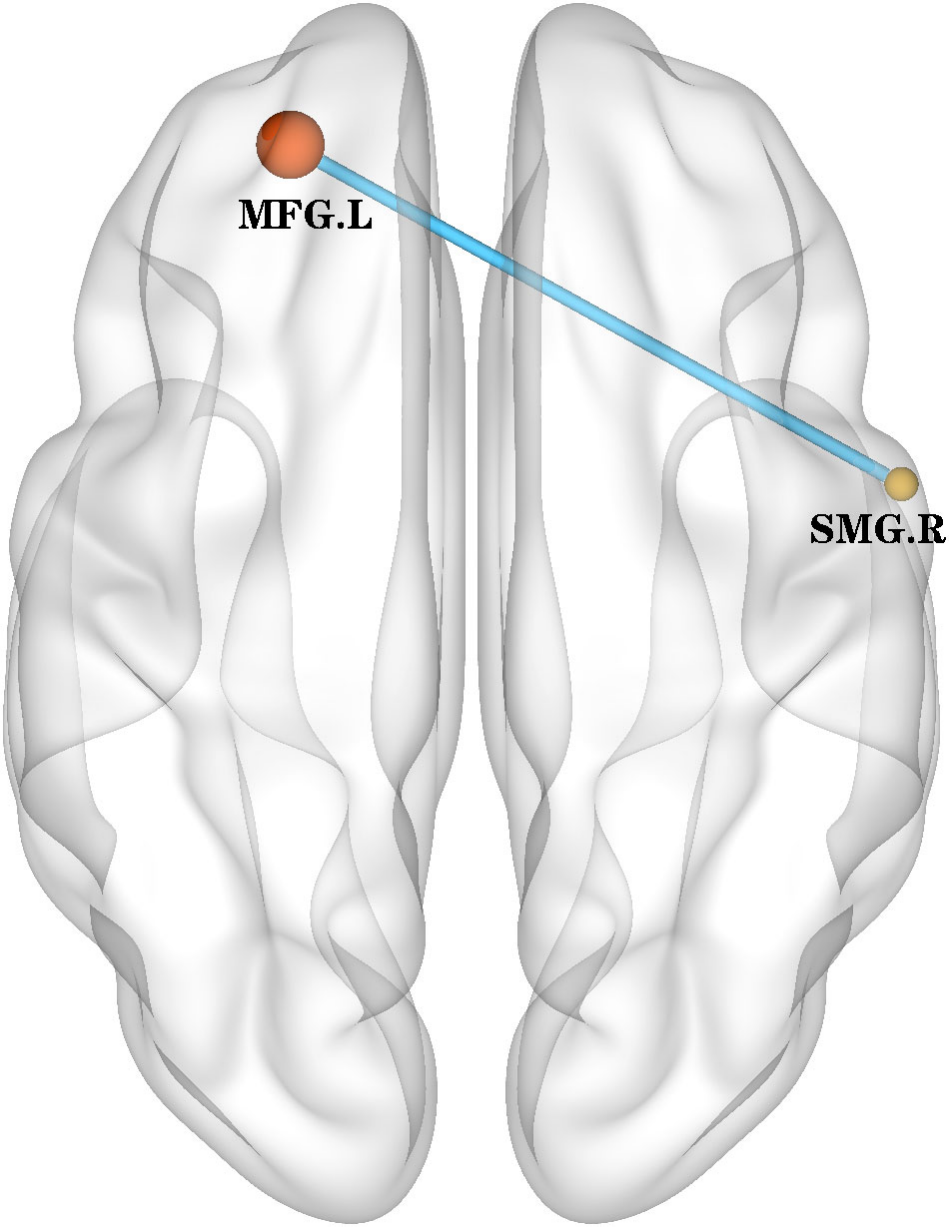
rsFC (using the overlapped 185 voxels as a seed) map between-group differences in the rsFC map. The rsFC between the right supramarginal gyrus and the left middle frontal gyrus was decreased. rsFC, resting-state functional connectivity; SMG.R, right Supramarginal gyrus; MFG.L, left middle frontal gyrus.

The only overlapping region exhibiting concurrent ALFF and ReHo abnormalities in BD patients was located in the right supramarginal gyrus (185 voxels). Using this cluster as the seed (**Supplementary Figure S1**), we found decreased rsFC between the right supramarginal gyrus and the left middle frontal gyrus (**Table 4**, **Figure 3**).

**Table 4:** Between-group differences in the rsFC analyses between the patients with BD and the healthy controls.

Seed		Brain region	Hemisphere	MNI coordinates	Peak T values	Cluster size	Cluster-level *P*_FWE_
185 voxels in the Supramarginal gyrus overlapped across ALFF and ReHo	Patients <Controls	Middle frontal gyrus	Left	-24,45,15	4.7608	908	<0.001

BD, bipolar disorder; ALFF, Amplitude of low-frequency fluctuation; ReHo, regional homogeneity; MNI, Montreal Neurological Institute

### 3.4 VBM analysis

Whole-brain analysis revealed that in the BD group, the gray matter volume in the left cerebellum was significantly reduced (**Supplementary Table S2**, **Supplementary Figure S2**). No regions showed increased gray matter volume.

### 3.5 Correlation analysis

We did not find a significant statistical correlation between the abnormal brain regions of ALFF and ReHo in the BD group and the total scores of HAMD, HAMA, and YMRS (all P > 0.017).

## 4 Discussion

In this article, we used two main functional indicators (ALFF and ReHo) to measure the spontaneous activities of local activity connections to the network and functional connections. Our results show that in patients diagnosed with BD, significant increases in ALFF were observed in the left posterior cingulate gyrus and the right parahippocampal gyrus (key nodes in the DMN). In the BD group, increased ALFF was observed in both the right middle frontal gyrus and the right supramarginal gyrus, suggesting a possible compensatory mechanism in these regions. However, ReHo was significantly decreased in several FPN-related regions, including the left superior frontal gyrus, right middle frontal gyrus, right inferior temporal gyrus, and bilateral parietal gyrus, suggesting a decline in their internal functional integration ability. Furthermore, using the overlapping region of the right supramarginal gyrus (the key node in the FPN) as the seed point of rsFC, we found that the rsFC within the FPN decreased, specifically manifested as the reduction of rsFC between the right supramarginal gyrus and the left middle frontal gyrus. Furthermore, we found that the gray matter volume in the posterior cerebellar lobe of BD patients was atrophied.

We used two functional indicators and found that compared with the healthy control group, the ALFF and ReHo values of BD patients in the DMN were increased. The increase of ALFF indicates an increase in local BOLD signal fluctuations (28, 29). The increase of ReHo indicates an increase in the synchronization ability of the related voxels (30). Most previous studies have suggested that patients with BD mainly present with functional abnormalities in the inferior frontal gyrus, middle frontal gyrus, anterior central gyrus and insula, mainly belonging to FPN and DMN (31–34). These results are consistent with those of our study. All in all, past and present studies have repeatedly emphasized the importance of DMN and FPN during the stable period of BD, which may indicate that they are involved in this pathological process. Previous studies have found that the low connectivity between the anterior and posterior DMN in manic BD patients and HC patients may be related to the attention pattern of over-focusing on external stimuli at the expense of internal reflexes (35). However, using independent component analysis have found that there is no difference in rsFC of DMN between BD patients and HC patients during the clinical remission period (36), and even there is no low connectivity in BD patients in remission (37, 38). This inconsistency might be caused by the heterogeneity of the samples, such as different types of BD or histories of mental illness. Abnormalities within the DMN are common, but they are reversed between the acute state and the remission state, which means that DMN injury is a characteristic of this disease and has different effects on BD individuals under different emotional states.

Subsequent rsFC analysis revealed that the connectivity of the left superior frontal gyrus and the left middle frontal gyrus (key nodes within the FPN network) in BD patients was low. Research reports indicate that compared with the control group, FC in the FPN of BD patients is reduced (39), which is consistent with our research results. However, Cipriani and Lu et al. found that, when it comes to the relationship between FPN and other networks, different studies gave completely opposite results. During the mitigation period, there was no significant difference between FPN and other networks (40, 41). However, a meta-analysis by Gong et al. observed low connectivity between FPN and DMN (42). On the contrary, Favre et al. reported an increase in the connection strength between FPN and DMN in BD with normal mood (43). The connection between FPN and DMN has not only been strengthened but also weakened. Our research has also found functional abnormalities of DMN, but no connection abnormalities between FPN and DMN have been found. This indicates that the inconsistent results may stem from the differences in the samples (such as BD subtypes, emotional states). In the future, a large amount of data will be needed for classification to further verify the results under different subtypes and different emotional states.

Whole-brain analysis revealed that BD presented a reduction in gray matter volume in the cerebellum. Previous studies have found that the volume of gray matter in the right cerebellum of BD patients is reduced (44), which is consistent with our research results. A topographic meta-analysis method used in functional neuroimaging studies indicates that this posterior cerebellar hemisphere region may be involved in higher-level tasks, including language and executive functions. With the activation of the posterior worm, the Crus I region, as part of the limbic circuit of the cerebellum, is also associated with emotion processing (45). A functional neuroimaging study on the cerebellum has shown that the reduction of FC in the cerebellum in BD patients involves the prefrontal lobe, cingulate gyrus, parietal lobe, temporal lobe, occipital bone and thalamic regions (46). Our study found that the ReHo of the left superior frontal gyrus, left parietal gyrus, right parietal gyrus, right middle frontal gyrus and right middle temporal gyrus was significantly reduced. This might be a functional disorder caused by cerebellar atrophy, especially in the context of rapid neural development during adolescence.

Previous studies have indicated that the cerebellum can be functionally subdivided into distinct regions based on its connectivity with the cerebrum. The posterior lobe of the cerebellum is associated with cognition and is connected to associative areas such as the prefrontal cortex, whereas the anterior cerebellum may modulate sensorimotor cortical activity. Given the uniform cytoarchitecture of the cerebellar cortex across lobules (47), it has been proposed that the cerebellum regulates both sensorimotor and associative cortical regions in a comparable manner. Similar to lesions in sensorimotor areas of the cerebellum, which lead to motor impairments (48), abnormalities in cerebellar regions involved in executive function and emotional regulation may contribute to cognitive dysfunctions. This concept has been proposed to explain symptoms of psychiatric disorders such as schizophrenia and mood symptoms (48, 49).

A previous meta-analysis of numerous neuroimaging studies revealed aberrant functional connectivity between the DMN and the FPN, accompanied by gray matter alterations within these networks. Reduced gray matter volume suggests potential neuronal loss, providing a biological basis for the observed abnormalities in functional connectivity (7). It is well-established that structural impairment inevitably leads to functional deficits within neural circuits. Building on earlier research in BD demonstrating abnormal functional connectivity between the cerebellum and the frontal and temporal lobes as well as the thalamus, we hypothesize that cerebellar gray matter atrophy may share underlying neural mechanisms with the functional alterations observed between the DMN and FPN. This structure-function coupling might be an important biomarker for the occurrence of BD. In the future, it is necessary to combine the development of neuroscience to explore whether these abnormalities are related to the asynchronous development of the brain during adolescence.

In the analysis of clinical scales, we did not observe any significant correlations. Previous studies have indicated differences in gender and subtypes among patients with BD) (50, 51). We speculate that this may be related to disease subtypes and patient gender. However, as we did not specifically investigate gender and disease subtypes, this represents a limitation of the present study.

Our study has several limitations that should be acknowledged. Firstly, there is heterogeneity in the emotional state of patients. In the future, more patients with the same emotional state need to be recruited to determine whether there are specific changes in the classification of disease subtypes. Secondly, and the use of drugs has not been strictly controlled, which may have a certain impact on the results. In future research, the types and dosages of drugs should be strictly controlled. Thirdly, We observed some misalignment in the image registration for part of the data, and future studies need to apply stricter registration standards to further validate the results.

## 5 Conclusions

This study provides novel insights into the neural underpinnings of adolescent bipolar disorder by combining ALFF, ReHo, rsFC, and GMV analyses. We demonstrated functional hyperactivity in DMN and limbic-related regions and reduced functional integration within the FPN. Furthermore, cerebellar gray matter atrophy may underlie broader functional disturbances observed in BD. Together, these multimodal findings support the notion that both functional and structural abnormalities are critical to the pathophysiology of adolescent BD. These alterations may serve as potential imaging biomarkers for early identification and intervention.

## Data Availability

The raw data supporting the conclusions of this article will be made available by the authors, without undue reservation.

## References

[1] Grande I Berk M Birmaher B Vieta E. Bipolar disorder. Lancet. (2016) 387:1561–72. doi: 10.1016/S0140-6736(15)00241-X, PMID: 26388529

[2] Alonso-Lana S Goikolea JM Bonnin CM Sarró S Segura B Amann BL. Structural and functional brain correlates of cognitive impairment in euthymic patients with bipolar disorder. PloS One. (2016) 11:e0158867. doi: 10.1371/journal.pone.0158867, PMID: 27448153 PMC4957815

[3] Bolton S Warner J Harriss E Geddes J Saunders KEA. Bipolar disorder: Trimodal age-at-onset distribution. Bipolar Disord. (2021) 23:341–56. doi: 10.1111/bdi.13016, PMID: 33030292 PMC8359178

[4] Phillips ML Swartz HA. A critical appraisal of neuroimaging studies of bipolar disorder: toward a new conceptualization of underlying neural circuitry and a road map for future research. Am J Psychiatry. (2014) 171:829–43. doi: 10.1176/appi.ajp.2014.13081008, PMID: 24626773 PMC4119497

[5] Townsend J Altshuler LL. Emotion processing and regulation in bipolar disorder: a review. Bipolar Disord. (2012) 14:326–39. doi: 10.1111/j.1399-5618.2012.01021.x, PMID: 22631618

[6] Vargas C López-Jaramillo C Vieta E. A systematic literature review of resting state network–functional MRI in bipolar disorder. J Affect Disord. (2013) 150:727–35. doi: 10.1016/j.jad.2013.05.083, PMID: 23830141

[7] Sha Z Wager TD Mechelli A He Y. Common dysfunction of large-scale neurocognitive networks across psychiatric disorders. Biol Psychiatry. (2019) 85:379–88. doi: 10.1016/j.biopsych.2018.11.011, PMID: 30612699

[8] Perry A Roberts G Mitchell PB Breakspear M. Connectomics of bipolar disorder: a critical review, and evidence for dynamic instabilities within interoceptive networks. Mol Psychiatry. (2019) 24:1296–318. doi: 10.1038/s41380-018-0267-2, PMID: 30279458 PMC6756092

[9] Narvacan K Treit S Camicioli R Martin W Beaulieu C. Evolution of deep gray matter volume across the human lifespan. Hum Brain Mapp. (2017) 38:3771–90. doi: 10.1002/hbm.23604, PMID: 28548250 PMC6867004

[10] Geary DC. Evolutionary perspective on sex differences in the expression of neurological diseases. Prog Neurobiol. (2019) 176:33–53. doi: 10.1016/j.pneurobio.2018.06.001, PMID: 29890214

[11] Whitwell JL. Voxel-based morphometry: an automated technique for assessing structural changes in the brain. J Neurosci. (2009) 29:9661–4. doi: 10.1523/jneurosci.2160-09.2009, PMID: 19657018 PMC6666603

[12] Lu X Zhong Y Ma Z Wu Y Fox PT Zhang N. Structural imaging biomarkers for bipolar disorder: Meta-analyses of whole-brain voxel-based morphometry studies. Depress Anxiety. (2019) 36:353–64. doi: 10.1002/da.22866, PMID: 30475436

[13] Späti J Hänggi J Doerig N Ernst J Sambataro F Brakowski J. Prefrontal thinning affects functional connectivity and regional homogeneity of the anterior cingulate cortex in depression. Neuropsychopharmacology. (2015) 40:1640–8. doi: 10.1038/npp.2015.8, PMID: 25598428 PMC4915268

[14] Van Tol MJ Li M Metzger CD Hailla N Horn DI Li W. Local cortical thinning links to resting-state disconnectivity in major depressive disorder. Psychol Med. (2014) 44:2053–65. doi: 10.1017/s0033291713002742, PMID: 24176247

[15] Zhang Y Zheng J Fan X Guo X Guo W Yang G. Dysfunctional resting-state connectivities of brain regions with structural deficits in drug-naive first-episode schizophrenia adolescents. Schizophr Res. (2015) 168:353–9. doi: 10.1016/j.schres.2015.07.031, PMID: 26281967

[16] Doucet GE He X Sperling M Sharan A Tracy JI. Gray matter abnormalities in temporal lobe epilepsy: relationships with resting-state functional connectivity and episodic memory performance. PloS One. (2016) 11:e0154660. doi: 10.1371/journal.pone.0154660, PMID: 27171178 PMC4865085

[17] Canu E Agosta F Sarasso E Volontè MA Basaia S Stojkovic T. Brain structural and functional connectivity in Parkinson's disease with freezing of gait. Hum Brain Mapp. (2015) 36:5064–78. doi: 10.1002/hbm.22994, PMID: 26359798 PMC6869160

[18] Zarp Petersen J Varo C Skovsen CF Ott CV Kjaerstad HL Vieta E. Neuronal underpinnings of cognitive impairment in bipolar disorder: A large data-driven functional magnetic resonance imaging study. Bipolar Disord. (2022) 24:69–81. doi: 10.1111/bdi.13100, PMID: 33955648

[19] Kollmann B Yuen K Scholz V Wessa M. Cognitive variability in bipolar I disorder: A cluster-analytic approach informed by resting-state data. Neuropharmacology. (2019) 156:107585. doi: 10.1016/j.neuropharm.2019.03.028, PMID: 30914304

[20] Chakrabarty T Torres IJ Su WW Sawatzky R Keramatian K Yatham LN. Cognitive subgroups in first episode bipolar I disorder: Relation to clinical and brain volumetric variables. Acta Psychiatr Scand. (2021) 143:151–61. doi: 10.1111/acps.13245, PMID: 33089491

[21] Njau S Townsend J Wade B Hellemann G Bookheimer S Narr K. Neural subtypes of euthymic bipolar I disorder characterized by emotion regulation circuitry. Biol Psychiatry Cognit Neurosci Neuroimaging. (2020) 5:591–600. doi: 10.1016/j.bpsc.2020.02.011, PMID: 32513391

[22] Kjærstad HL Damgaard V Knudsen GM Vinberg M Kessing LV Macoveanu J. Neural underpinnings of emotion regulation subgroups in remitted patients with recently diagnosed bipolar disorder. Eur Neuropsychopharmacol. (2022) 60:7–18. doi: 10.1016/j.euroneuro.2022.04.010, PMID: 35550452

[23] Li WH Tang LR Wang M Wang JN Guo T He Q. Altered gray matter volume and functional connectivity in medial orbitofrontal cortex of bulimia nervosa patients: A combined VBM and FC study. Front Psychiatry. (2022) 13:963092. doi: 10.3389/fpsyt.2022.963092, PMID: 36061303 PMC9437330

[24] Yan CG Wang XD Zuo XN Zang YF. DPABI: data processing & Analysis for (Resting-state) brain imaging. Neuroinformatics. (2016) 14:339–51. doi: 10.1007/s12021-016-9299-4, PMID: 27075850

[25] Friston KJ Williams S Howard R Frackowiak RS Turner R. Movement-related effects in fMRI time-series. Magn Reson Med. (1996) 35:346–55. doi: 10.1002/mrm.1910350312, PMID: 8699946

[26] Power JD Barnes KA Snyder AZ Schlaggar BL Petersen SE. Spurious but systematic correlations in functional connectivity MRI networks arise from subject motion. Neuroimage. (2012) 59:2142–54. doi: 10.1016/j.neuroimage.2011.10.018, PMID: 22019881 PMC3254728

[27] Power JD Mitra A Laumann TO Snyder AZ Schlaggar BL Petersen SE. Methods to detect, characterize, and remove motion artifact in resting state fMRI. Neuroimage. (2014) 84:320–41. doi: 10.1016/j.neuroimage.2013.08.048, PMID: 23994314 PMC3849338

[28] Zou QH Zhu CZ Yang Y Zuo XN Long XY Cao QJ. An improved approach to detection of amplitude of low-frequency fluctuation (ALFF) for resting-state fMRI: fractional ALFF. J Neurosci Methods. (2008) 172:137–41. doi: 10.1016/j.jneumeth.2008.04.012, PMID: 18501969 PMC3902859

[29] Wang JJ Chen X Sah SK Zeng C Li YM Li N. Amplitude of low-frequency fluctuation (ALFF) and fractional ALFF in migraine patients: a resting-state functional MRI study. Clin Radiol. (2016) 71:558–64. doi: 10.1016/j.crad.2016.03.004, PMID: 27055741

[30] Zang Y Jiang T Lu Y He Y Tian L. Regional homogeneity approach to fMRI data analysis. Neuroimage. (2004) 22:394–400. doi: 10.1016/j.neuroimage.2003.12.030, PMID: 15110032

[31] Sun F Liu Z Yang J Fan Z Xi C Cheng P. Shared and distinct patterns of dynamical degree centrality in bipolar disorder across different mood states. Front Psychiatry. (2022) 13:941073. doi: 10.3389/fpsyt.2022.941073, PMID: 35966464 PMC9364672

[32] Li M Das T Deng W Wang Q Li Y Zhao L. Clinical utility of a short resting-state MRI scan in differentiating bipolar from unipolar depression. Acta Psychiatr Scand. (2017) 136:288–99. doi: 10.1111/acps.12752, PMID: 28504840

[33] Russo D Martino M Magioncalda P Inglese M Amore M Northoff G. Opposing changes in the functional architecture of large-scale networks in bipolar mania and depression. Schizophr Bull. (2020) 46:971–80. doi: 10.1093/schbul/sbaa004, PMID: 32047938 PMC7342167

[34] Skåtun KC Kaufmann T Tønnesen S Biele G Melle I Agartz I. Global brain connectivity alterations in patients with schizophrenia and bipolar spectrum disorders. J Psychiatry Neurosci. (2016) 41:331–41. doi: 10.1503/jpn.150159, PMID: 26854755 PMC5008922

[35] Friston KJ Frith CD Liddle PF Dolan RJ Lammertsma AA Frackowiak RS. The relationship between global and local changes in PET scans. J Cereb Blood Flow Metab. (1990) 10:458–66. doi: 10.1038/jcbfm.1990.88, PMID: 2347879

[36] Eklund A Nichols TE Knutsson H. Cluster failure: Why fMRI inferences for spatial extent have inflated false-positive rates. Proc Natl Acad Sci U S A. (2016) 113:7900–5. doi: 10.1073/pnas.1602413113, PMID: 27357684 PMC4948312

[37] Fernández-Corcuera P Salvador R Monté GC Salvador Sarró S Goikolea JM Amann B. Bipolar depressed patients show both failure to activate and failure to de-activate during performance of a working memory task. J Affect Disord. (2013) 148:170–8. doi: 10.1016/j.jad.2012.04.009, PMID: 22854099

[38] Smith SM Nichols TE. Threshold-free cluster enhancement: addressing problems of smoothing, threshold dependence and localisation in cluster inference. Neuroimage. (2009) 44:83–98. doi: 10.1016/j.neuroimage.2008.03.061, PMID: 18501637

[39] Liang YS Zhou SZ Zhang YJ Cai XL Wang Y Cheung EFC. Altered empathy-related resting-state functional connectivity in patients with bipolar disorder. Eur Arch Psychiatry Clin Neurosci. (2022) 272:839–48. doi: 10.1007/s00406-021-01305-4, PMID: 34282469

[40] Syan SK Minuzzi L Smith M Allega OR Hall GB Frey BN. Resting state functional connectivity in women with bipolar disorder during clinical remission. Bipolar Disord. (2017) 19:97–106. doi: 10.1111/bdi.12469, PMID: 28258639

[41] Yip SW Mackay CE Goodwin GM. Increased temporo-insular engagement in unmedicated bipolar II disorder: an exploratory resting state study using independent component analysis. Bipolar Disord. (2014) 16:748–55. doi: 10.1111/bdi.12206, PMID: 24725219

[42] Gong J Wang J Chen P Qi Z Luo Z Wang J. Large-scale network abnormality in bipolar disorder: A multimodal meta-analysis of resting-state functional and structural magnetic resonance imaging studies. J Affect Disord. (2021) 292:9–20. doi: 10.1016/j.jad.2021.05.052, PMID: 34087634

[43] Favre P Baciu M Pichat C Bougerol T Polosan M. fMRI evidence for abnormal resting-state functional connectivity in euthymic bipolar patients. J Affect Disord. (2014) 165:182–9. doi: 10.1016/j.jad.2014.04.054, PMID: 24882198

[44] Kim D Cho HB Dager SR Yurgelun-Todd DA Yoon S Lee JH. Posterior cerebellar vermal deficits in bipolar disorder. J Affect Disord. (2013) 150:499–506. doi: 10.1016/j.jad.2013.04.050, PMID: 23769608 PMC5510461

[45] Stoodley CJ Schmahmann JD. Evidence for topographic organization in the cerebellum of motor control versus cognitive and affective processing. Cortex. (2010) 46:831–44. doi: 10.1016/j.cortex.2009.11.008, PMID: 20152963 PMC2873095

[46] Cattarinussi G Di Giorgio A Sambataro F. Cerebellar dysconnectivity in schizophrenia and bipolar disorder is associated with cognitive and clinical variables. Schizophr Res. (2024) 267:497–506. doi: 10.1016/j.schres.2024.03.039, PMID: 38582653

[47] Zhou Y Shi L Cui X Wang S Luo X. Functional connectivity of the caudal anterior cingulate cortex is decreased in autism. PloS One. (2016) 11:e0151879. doi: 10.1371/journal.pone.0151879, PMID: 26985666 PMC4795711

[48] Schmahmann JD. Disorders of the cerebellum: ataxia, dysmetria of thought, and the cerebellar cognitive affective syndrome. J Neuropsychiatry Clin Neurosci. (2004) 16:367–78. doi: 10.1176/jnp.16.3.367, PMID: 15377747

[49] Andreasen NC Pierson R. The role of the cerebellum in schizophrenia. Biol Psychiatry. (2008) 64:81–8. doi: 10.1016/j.biopsych.2008.01.003, PMID: 18395701 PMC3175494

[50] Sun H Yan R Chen Z Wang X Xia Y Hua L. Common and disease-specific patterns of functional connectivity and topology alterations across unipolar and bipolar disorder during depressive episodes: a transdiagnostic study. Transl Psychiatry. (2025) 15:58. doi: 10.1038/s41398-025-03282-x, PMID: 39966397 PMC11836414

[51] Colic L Clark A Sankar A Rathi DJ Goldman DA Kim JA. Gender-related association among childhood maltreatment, brain structure and clinical features in bipolar disorder. Eur Neuropsychopharmacol. (2022) 63:35–46. doi: 10.1016/j.euroneuro.2022.07.186, PMID: 36037590 PMC9593266

