# Supplementary material for: Multimodal MRI study of gray matter and functional connectivity abnormalities in adolescents with bipolar disorder
Source: Front Psychiatry. 2025 Oct 16;16:1664729. doi: 10.3389/fpsyt.2025.1664729 (PMC12573138; doi:10.3389/fpsyt.2025.1664729)
Supplement: Supplementary file 1 [file SupplementaryFile1.docx]

Supplementary Material

# Supplementary Figures and Tables

| **TableS1 Between-group differences in the ALFF and ReHo analyses between the patients with BD and the healthy controls** | | | | | | | |
| --- | --- | --- | --- | --- | --- | --- | --- |
|  |  | **Brain region** | **Hemisphere** | **MNI coordinates** | **Peak T values** | **Cluster size** | **Cluster-level *P*_FWE_** |
| **ALFF** | Patients > Controls | Supramarginal gyrus | Right | 48,-21,27 | 6.1856 | 468 | <0.001 |
| **REHO** | Patients > Controls | Supramarginal gyrus | Right | 48,-21,27 | 5.1411 | 229 | 0.001 |
| BD: bipolar disorder; ALFF: Amplitude of low-frequency fluctuation; ReHo: regional homogeneity; MNI: Montreal Neurological Institute | | | | | | | |

| **TableS2 Between-group differences in the Grey Matter analyses between the patients with BD and the healthy controls** | | | | | | |
| --- | --- | --- | --- | --- | --- | --- |
|  | **Brain region** | **Hemisphere** | **MNI coordinates** | **Peak T values** | **Cluster size** | **Cluster-level *P*_FWE_** |
| **Patients <Controls** | Cerebellum_Inferior_9 | Left | 0,-51,-62 | 4.9747 | 1332 | 0.03 |
| BD: bipolar disorder; MNI: Montreal Neurological Institute. | | | | | | |

**FigureS1 The overlapping region of ALFF and ReHo abnormalities in BD patients**

**
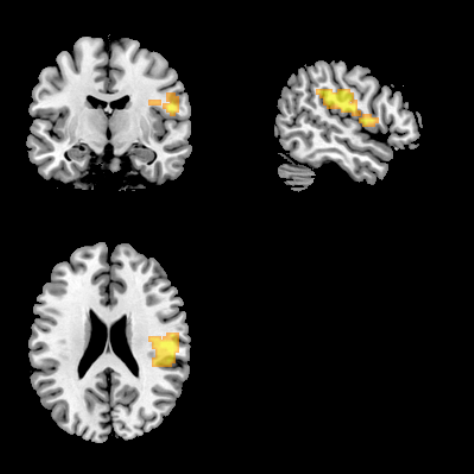
**

**FigureS2 Between-group differences in the Gray Matter analyses between the patients with bipolar disorder and the healthy controls (bipolar disorder > health controls)**

**
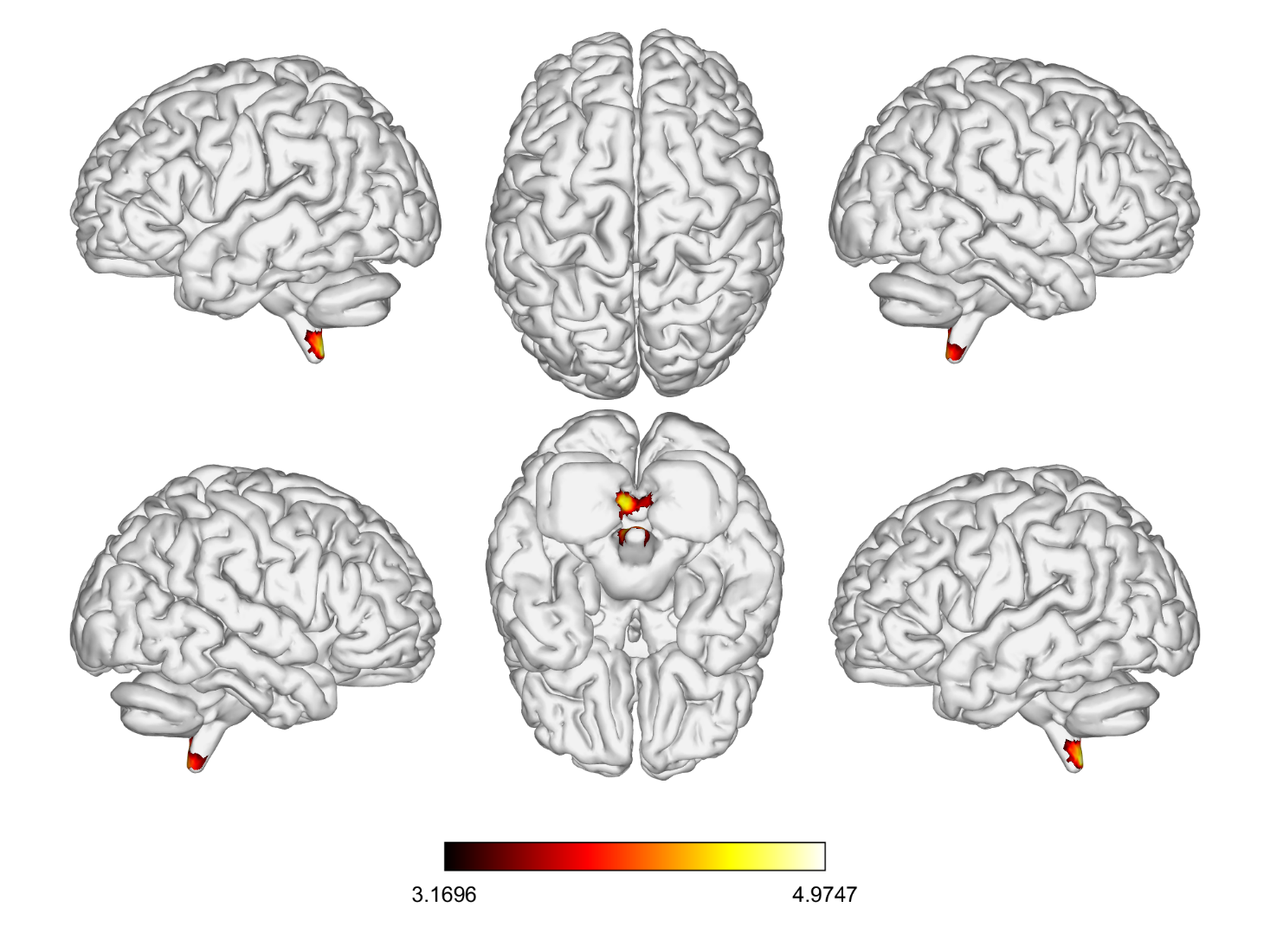
**
